# Supplementary material for: What Sustains Mask-Wearing Behavior among Elders in a Rural Community in the Post-COVID-19 Era: An Exploratory Mixed-Methods Study
Source: Behav Sci (Basel). 2023 Aug 11;13(8):678. doi: 10.3390/bs13080678 (PMC10451204; doi:10.3390/bs13080678)
Supplement: Supplementary file 1 [file behavsci-13-00678-s001.zip › behavsci-2443816-supplementary.pdf]

Supplementary File

Table S1. Exploratory factor analysis of mask wearing questionnaire. Page 2-3
Table S2. Thematic analysis of participants' themes, subthemes, and codes Page 4
Table S3. Factors influencing mask-wearing among elders in a rural community in central Thailand Page 5-7

Figure S1. Full questionnaire used in the study. Page 8-10

Supplementary Table S1. Exploratory factor analysis of mask wearing questionnaire

Questionnaire items	Domains								
	**Domain 1**	**Domain 2**	**Domain 3**	**Domain 4**	**Domain 5**	**Domain 6**	**Domain 7**	**Domain 8**	**Domain 9**
1. Currently, I am willing to wear a face mask	**0.862**	0.191	0.010	0.092	-0.008	0.193	-0.083	-0.037	0.204
2. I am willing to spend money on face masks	**0.717**	0.144	0.017	0.079	0.025	0.064	-0.125	0.076	0.119
3. I always suggest that others wear masks	0.366	0.190	-0.182	0.210	0.022	-0.263	-0.205	0.176	0.024
4. I am willing to wear a mask, despite the official announcement not to enforce mask wearing	**0.854**	0.123	0.082	0.153	0.006	0.122	-0.033	0.166	0.095
5. After an official announcement not to enforce face mask-wearing; how long do you plan to wear a mask?	**0.560**	0.322	0.058	0.172	0.159	0.079	-0.105	0.109	-0.080
6. In the past month, how frequently did you change your face mask?	0.078	**0.659**	0.022	0.144	-0.017	0.050	0.144	0.237	0.255
7. In the past month, did you wear a mask?	0.145	**0.830**	-0.063	0.138	0.025	-0.057	0.103	0.083	0.124
8. How frequently did you wear a mask weekly?	0.213	**0.467**	-0.036	0.213	-0.070	0.138	0.050	0.032	-0.148
9. I always bring a mask every time I go outside	0.201	**0.514**	0.010	0.029	0.018	0.027	0.048	0.297	0.100
10. I am familiar with mask-wearing	0.243	**0.509**	-0.004	0.266	0.085	0.204	-0.027	0.172	-0.081
11. I believe that COVID-19 is still spreading throughout my community	0.043	0.083	-0.047	0.142	-0.005	0.111	-0.072	0.041	**0.465**
12. I believe that COVID-19 can reemerge in the future	0.259	-0.047	0.078	0.099	0.146	0.078	0.043	0.122	**0.396**
13. I still feel anxious about being infected with COVID-19	0.301	0.170	0.026	0.134	0.080	0.088	-0.141	0.203	**0.386**
14. I feel anxious or disgusted when someone not wearing a mask gets close to me	0.205	0.147	0.028	0.111	-0.017	-0.036	-0.212	**0.433**	0.002
15. I wear a mask to protect myself from air pollution	0.103	0.239	-0.105	0.090	-0.003	0.134	-0.005	**0.616**	0.042
16. I wear a mask to protect myself against other respiratory infections apart from COVID-19	-0.007	0.167	-0.207	0.149	0.027	0.059	0.096	**0.627**	0.252
17*. I trusted that Andrographis paniculata could effectively prevent COVID-19, so I reduced my mask-wearing.	0.002	0.035	**0.757**	-0.003	0.067	-0.001	-0.045	0.001	0.045
18*. I trusted that practices such as eating cooked food, using serving spoons, and washing hands could effectively prevent COVID-19, so I reduced my mask-wearing.	0.085	0.029	**0.944**	0.012	0.061	0.076	-0.007	-0.078	0.028
19*. I trusted that cloth face masks could effectively prevent COVID-19, so I reduced my mask-wearing	0.036	-0.063	**0.398**	0.069	0.124	0.088	-0.021	-0.075	-0.034
20. I believe in the effectiveness of face masks for COVID-19 protection.	0.126	0.180	0.012	**0.757**	-0.057	-0.031	0.140	0.067	0.337
21. I believe that mask-wearing helps reduce the spread of COVID-19	0.228	0.167	0.004	**0.725**	0.014	0.043	0.053	0.159	0.155
22. Wearing a face mask improves my self-esteem	0.143	0.227	0.166	**0.467**	-0.048	0.084	-0.073	0.185	-0.013
23. I believe that using a face mask makes me look better	0.041	0.170	-0.127	0.287	-0.069	0.223	0.230	0.011	0.089
24*. I feel uncomfortable when wearing a mask	0.121	0.055	0.015	0.098	0.162	**0.742**	-0.132	0.013	0.036
25*. Wearing a mask impairs my work productivity	0.194	0.023	0.042	0.010	0.147	**0.565**	-0.080	0.039	0.046
26*. Wearing a mask can be an obstacle to communication	-0.008	0.105	0.143	0.023	0.128	**0.535**	-0.018	0.106	0.168

27. I wear a face mask because it is a social measure.	-0.074	0.085	-0.075	0.047	-0.136	-0.104	**0.744**	-0.052	-0.088
28. I wear a face mask because others are also wearing them.	-0.209	0.172	0.007	0.117	-0.112	-0.136	**0.736**	0.014	-0.015
29. I wear a face mask to conceal my poor oral hygiene	0.142	0.074	-0.286	0.173	-0.039	0.020	-0.265	0.231	0.071
30*. I don't have enough high-quality face masks to meet the standard.	0.003	-0.027	0.153	-0.007	0.351	0.099	-0.106	-0.039	-0.056
31*. The high price is affecting my decision to purchase a face mask.	0.038	-0.017	0.042	-0.088	**0.634**	0.134	-0.028	0.071	0.093
32*. I don't have enough money to afford using a face mask daily.	0.136	-0.006	0.083	-0.001	**0.763**	0.108	-0.009	0.092	-0.086
33*. I think that buying face masks increases my financial burden.	-0.054	0.094	0.015	0.024	**0.708**	0.041	-0.083	-0.126	0.201
**Eigenvalues**	**6.40**	**3.32**	**2.29**	**2.00**	**1.59**	**1.50**	**1.44**	**1.23**	**1.08**

Extraction Method: Maximum Likelihood. Rotation Method: Varimax with Kaiser Normalization. Rotation converged in 6 iterations.

* Negative questions

Supplementary Table S2. Thematic analysis of participants' themes, subthemes, and codes

Theme	Sub-theme	Code
Facilitators/Motivators	Perceived benefits of mask-wearing	disease prevention; confidence in the effectiveness of the mask; reducing COVID-19 infection rate; preventing the spread of respiratory infections; protection from dust, protection from PM2.5
	Perceived threat of COVID-19	COVID-19 might return; visitors might carry COVID-19; having respiratory symptoms; uncertainty about other people; fear of contracting COVID-19; COVID-19 can lead to hospitalization
	Mask wearing enhancing attractiveness and self-confidence	increase self-confidence; hide poor oral hygiene; feeling more confident after wearing a mask; looking cleaner after wearing a mask
	Social norms and new-normal	wearing mask in crowded areas; necessary for leaving the house; other people wear the mask; there are many people around; familiarity and habit; disgusted by those not wearing a mask; role model in wearing a mask
Barriers/Obstacles	Misconceptions about COVID-19 prevention tools	*Andrographis paniculata* could prevent COVID-19; eating hot food could prevent COVID-19; eating with middle spoon and washing hands is enough to prevent COVID-19; cloth face mask is as effective as surgical face mask
	Perceived barriers to mask-wearing	hinders communication; suffocating; difficult to smoke; allergic to masks; blocking makeup; hot and humid; hinders working; more prone to fatigue
	Resources to afford face masks	no standard masks available; unable to afford to buy masks; shortage of masks; expensive

Supplementary Table S3. Factors influencing mask-wearing among elders in a rural community in central Thailand

Questions	Mask adherance	5 *n* (%)	4 *n* (%)	3 *n* (%)	2 *n* (%)	1 *n* (%)	λ
Willingness to wear face masks							
**Will 1.** Currently, I am willing to wear a face mask	Not adhering	13 (34.2)	1 (2.6)	8 (21.1)	2 (5.3)	14 (36.8)	0.86
	Adhering	108 (66.3)	10 (6.1)	7 (4.3)	3 (1.8)	35 (21.5)	
**Will 2.** I am willing to spend money on face masks	Not adhering	17 (44.7)	3 (7.9)	3 (7.9)	3 (7.9)	12 (31.6)	0.72
	Adhering	103 (63.2)	12 (7.4)	7 (4.3)	4 (2.5)	37 (22.7)	
**Will 3.** I am willing to wear a mask, despite the official announcement not to enforce mask wearing	Not adhering	18 (47.4)	1 (2.6)	3 (7.9)	4 (10.5)	12 (31.6)	0.85
	Adhering	111 (68.1)	10 (6.1)	7 (4.3)	2 (1.2)	33 (20.2)	
****Will 4.** After an official announcement not to enforce face mask-wearing; how long do you plan to wear a mask?	Not adhering	12 (31.6)	4 (10.5)	4 (10.5)	2 (5.3)	16 (42.1)	0.56
	Adhering	105 (64.4)	12 (7.4)	19 (11.7)	5 (3.1)	22 (13.5)	
Overall Willingness Likert score (Mean±SD)	Not adhering			3.07±1.50		**t**	***p*-value**
	Adhering			3.97±1.36		-3.610	<0.001
Mask wearing practice							
****Prac 1.** In the past month, did you wear a mask?	Not adhering	0	0	28 (100.0)	0	10 (100.0)	0.83
	Adhering	163 (100.0)	0	0	0	0	
****Prac 2.** In the past month, how frequently did you change your face mask?	Not adhering	2 (5.3)	5 (13.2)	10 (26.3)	11 (28.9)	10 (26.3)	0.66
	Adhering	62 (38.0)	22 (13.5)	38 (23.3)	39 (23.9)	2 (1.2)	
****Prac 3.** How frequently did you wear a mask weekly?	Not adhering	2 (5.3)	22 (57.9)	4 (10.5)	0 (0)	10 (26.3)	0.47
	Adhering	41 (25.2)	106 (65.0)	13 (8.0)	3 (1.8)	0 (0)	
**Prac 4.** I always bring a mask every time I go outside	Not adhering	24 (63.2)	3 (7.9)	1 (2.6)	2 (5.3)	8 (21.1)	0.51
	Adhering	149 (91.4)	7 (4.3)	3 (1.8)	2 (1.2)	2 (1.2)	
**Prac 5.** I am familiar with mask-wearing	Not adhering	14 (36.8)	2 (5.3)	5 (13.2)	4 (10.5)	13 (34.2)	0.51
	Adhering	133 (81.6)	9 (5.5)	2 (1.2)	3 (1.8)	16 (9.8)	
Overall mask wearing practice Likert score (Mean±SD)	Not adhering			2.93±1.19		**t**	***p*-value**
	Adhering			4.41±0.47		-12.331	<0.001
Perceived threat toward COVID-19							
**Threat 1.** I believe that COVID-19 is still spreading throughout my community	Not adhering	6 (15.8)	2 (5.3)	3 (7.9)	3 (7.9)	24 (63.2)	0.47
	Adhering	56 (34.4)	12 (7.4)	15 (9.2)	8 (4.9)	72 (44.2)	
**Threat 2.** I believe that COVID-19 can reemerge in the future	Not adhering	13 (34.2)	2 (5.3)	7 (18.4)	1 (2.6)	15 (39.5)	0.40
	Adhering	56 (34.4)	19 (11.7)	26 (16.0)	9 (5.5)	53 (32.5)	
**Threat 3.** I still feel anxious about being infected with COVID-19	Not adhering	5 (13.2)	1 (2.6)	4 (10.5)	0 (0)	28 (73.7)	0.39
	Adhering	64 (39.3)	6 (3.7)	19 (11.7)	10 (6.1)	64 (39.3)	
Overall perceived threat Likert score (Mean±SD)	Not adhering			2.25±1.12		**t**	***p*-value**
	Adhering			2.97±1.31		-3.106	<0.001
Alternative perceived threat aside from COVID-19							
**Alt threat 1.** I feel anxious or disgusted when someone not wearing a mask gets close to me	Not adhering	9 (23.7)	2 (5.3)	5 (13.2)	3 (7.9)	19 (50.0)	0.43
	Adhering	66 (40.5)	16 (9.8)	14 (8.6)	9 (5.5)	58 (35.6)	

**Alt threat 2**. I wear a mask to protect myself from air pollution	Not adhering	19 (50.0)	1 (2.6)	3 (7.9)	1 (2.6)	14 (36.8)	0.62
	Adhering	107 (65.6)	21 (12.9)	3 (1.8)	5 (3.1)	27 (16.6)	
**Alt threat 3.** I wear a mask to protect myself against other respiratory infections apart from COVID-19	Not adhering	13 (34.2)	0 (0)	2 (5.3)	0 (0)	23 (60.5)	0.63
	Adhering	82 (50.3)	15 (9.2)	12 (7.4)	2 (1.2)	52 (31.9)	
Overall alternative perceived threat Likert score (Mean±SD)	Not adhering			2.73±1.57		**t**	***p*-value**
	Adhering			3.56±1.22		-3.551	<0.001
Misconception in COVID-19 prevention tools							
***Mis 1.** I trusted that Andrographis paniculata could effectively prevent COVID-19, so I reduced my mask-wearing.	Not adhering	24 (63.2)	0 (0)	5 (13.2)	2 (5.3)	7 (18.4)	0.76
	Adhering	89 (54.6)	14 (8.6)	18 (11.0)	9 (5.5)	33 (20.2)	
***Mis 2**. I trusted that practices such as eating cooked food, using serving spoons, and washing hands could effectively prevent COVID-19, so I reduced my mask-wearing.	Not adhering	23 (60.5)	1 (2.6)	1 (2.6)	2 (5.3)	11 (28.9)	0.94
	Adhering	82 (50.3)	9 (5.5)	11 (6.7)	13 (8.0)	48 (29.4)	
***Mis 3.** I trusted that cloth face masks could effectively prevent COVID-19, so I reduced my mask-wearing	Not adhering	23 (60.5)	2 (5.3)	5 (13.2)	0 (0)	8 (21.1)	0.40
	Adhering	89 (54.6)	13 (8.0)	15 (9.2)	5 (3.1)	41 (25.2)	
Overall misconception Likert score (Mean±SD)	Not adhering			3.76±1.35		**t**	***p*-value**
	Adhering			3.58±1.38		0.727	0.234
Perceived benefits of face mask							
**Benefits 1.** I believe in the effectiveness of face masks for COVID-19 protection.	Not adhering	11 (28.9)	6 (15.8)	7 (18.4)	3 (7.9)	11 (28.9)	0.76
	Adhering	78 (47.9)	35 (21.5)	33 (20.2)	3 (1.8)	14 (8.6)	
**Benefits 2**. I believe that mask-wearing helps reduce the spread of COVID-19	Not adhering	16 (42.1)	4 (10.5)	5 (13.2)	3 (7.9)	10 (26.3)	0.73
	Adhering	107 (65.6)	21 (12.9)	17 (10.4)	2 (1.2)	16 (9.8)	
**Benefits 3.** Wearing a face mask improves my self-esteem	Not adhering	16 (42.1)	4 (10.5)	3 (7.9)	2 (5.3)	13 (34.2)	0.47
	Adhering	110 (67.5)	10 (6.1)	10 (6.1)	4 (2.5)	29 (17.8)	
Overall perceived benefits Likert score (Mean±SD)	Not adhering			3.21±1.47		**t**	***p*-value**
	Adhering			4.08±1.07		-4.193	<0.001
Discomfort when wearing a face mask							
***Discomfort 1.** I feel uncomfortable when wearing a mask	Not adhering	11 (28.9)	0 (0)	9 (23.7)	2 (5.3)	16 (42.1)	0.74
	Adhering	58 (35.6)	12 (7.4)	16 (9.8)	13 (8.0)	64 (39.3)	
***Discomfort 2**. Wearing a mask impairs my work productivity	Not adhering	16 (42.1)	1 (2.6)	3 (7.9)	2 (5.3)	16 (42.1)	0.57
	Adhering	72 (44.2)	10 (6.1)	10 (6.1)	16 (9.8)	55 (33.7)	
***Discomfort 3.** Wearing a mask can be an obstacle to communication	Not adhering	18 (47.4)	1 (2.6)	3 (7.9)	6 (15.8)	10 (26.3)	0.54
	Adhering	94 (57.7)	9 (5.5)	12 (7.4)	14 (8.6)	34 (20.9)	
Overall discomfort Likert score (Mean±SD)	Not adhering			2.98±1.44		**t**	***p*-value**
	Adhering			3.27±1.38		-1.132	0.130
Social norms							
**Social 1.** I wear a face mask because it is a social measure.	Not adhering	18 (47.4)	2 (5.3)	2 (5.3)	0 (0)	16 (42.1)	0.74
	Adhering	104 (63.8)	6 (3.7)	9 (5.5)	1 (0.6)	43 (26.4)	
**Social 2.** I wear a face mask because others are also wearing them.	Not adhering	15 (39.5)	1 (2.6)	3 (7.9)	1 (2.6)	18 (47.4)	0.74
	Adhering	100 (61.3)	5 (3.1)	9 (5.5)	3 (1.8)	46 (28.2)	
Overall social norms Likert score (Mean±SD)	Not adhering			3.00±1.69		**t**	***p*-value**
	Adhering			3.73±1.60		-2.498	0.007

Budget resources for affording face masks							
***Cost 1.** The high price is affecting my decision to purchase a face mask.	Not adhering	18 (47.4)	1 (2.6)	4 (10.5)	5 (13.2)	10 (26.3)	0.74
	Adhering	71 (43.6)	9 (5.5)	11 (6.7)	17 (10.4)	55 (33.7)	
***Cost 2**. I don’t have enough money to afford using a face mask daily.	Not adhering	23 (60.5)	1 (2.6)	3 (7.9)	1 (2.6)	10 (26.3)	0.57
	Adhering	95 (58.3)	14 (8.6)	10 (6.1)	13 (8.0)	31 (19.0)	
***Cost 3.** I think that buying face masks increases my financial burden.	Not adhering	12 (31.6)	2 (5.3)	2 (5.3)	3 (7.9)	19 (50.0)	0.54
	Adhering	65 (39.9)	13 (8.0)	12 (7.4)	15 (9.2)	58 (35.6)	
Overall budget resources Likert score (Mean±SD)	Not adhering		3.20±1.44			**t**	***p*-value**
	Adhering		3.34±1.42			-0.528	0.299

5= strongly agree, 4= agree, 3= mediocre, 2= disagree, 1= strongly disagree, λ= loading factor
*Negative questions: 5= strongly disagree, 4= disagree, 3= mediocre, 2= agree, 1= strongly agree
**Answers for the questions are included in the Supplementary Figure S1

Supplementary Figure S1

Case Record Form

Factors influencing mask-wearing among elders in a rural community in central Thailand

Demographic data

Nationality ____________ **Age** _______ years old **Marital status** ☐ Single ☐ Married ☐ Divorced ☐ Widow

Gender ☐ Male ☐ Female ☐ Others

Education

- ☐ Less than primary school
- ☐ Primary school
- ☐ Middle school
- ☐ High school
- ☐ Bachelor or associate's degree
- ☐ Master's degree or higher

Moo ☐ 8 ☐ 10 ☐ 13 ☐ 16

Health care

- ☐ Universal Health Coverage
- ☐ Government or State Enterprise Officer
- ☐ Social Security Scheme
- ☐ Paid

Smoking

- ☐ Everyday
- ☐ More than 3 times a week
- ☐ Less than 3 times a week
- ☐ Never

Family Income ☐,☐☐☐,☐☐☐ **baht/month**

Occupation

- ☐ Unemployed, Household
- ☐ Agriculturist
- ☐ Career, business owner
- ☐ Employee
- ☐ Government officer
- ☐ Student
- ☐ Others ....

Alcohol drinking

- ☐ 4 times or more per week
- ☐ 2-3 times per week
- ☐ 2-4 times per months
- ☐ Once a month or less
- ☐ Never

Underlying disease

- ☐ Hypertension
- ☐ Diabetes mellitus
- ☐ Dyslipidemia
- ☐ Chronic kidney disease
- ☐ Others ....

COVID-19 quiz		
**Is the following statement true?**	**True**	**False**
1. The following symptoms are relevant to COVID-19: fever, cough, runny nose, sore throat and phlegm. Associated symptoms such as diarrhea, fatigue, headache, muscle pain and dyspnea are also possible.		
2. ATK testing shouldn't be done every day. It should be done when suspected symptoms such as malaise, cough, sore throat and runny nose are noted.		
3. For those with COVID-19 ATK test positive, antiviral drugs are always needed.		
4. When you're infected with COVID-19, supportive medicine is suggested, such as antipyretics and mucolytics. Drinking a lot of water, using a tepid sponge and getting plenty of rest are also		

recommended.		
5. These interventions could prevent the spread of the COVID-19 virus: social distancing of about 1-2 meters, mask-wearing, and frequently washing your hands.		
6. You should see a doctor if you have severe symptoms such as a fever over 39 degrees Celsius or an oxygen saturation below 94%.		
7. After COVID-19 infection, once your COVID-19 ATK test is negative, you could live outside your home normally without wearing a face mask.		
8. Wearing a cloth face mask could prevent COVID-19 infection as effectively as a surgical face mask.		
9. Face masks should be changed daily.		
10. To save money, a face mask can be reused by switching the sides of the mask.		

Mask wearing behavior

In the past month, did you wear a mask?

- ☐ I always wore a face mask outside their home or when meeting others (5)
- ☐ sometimes wore a face mask outside their home or when meeting others (3)
- ☐ I didn't wear a face mask (1)

How frequently did you wear a mask weekly?

- ☐ Daily (5)
- ☐ More than 3 times a week (4)
- ☐ Less than 3 times a week (3)
- ☐ Once a week (2)
- ☐ Didn't wear a face mask (1)

After an official announcement not to enforce face mask-wearing; how long do you plan to wear a mask?

- ☐ Immediately (1)
- ☐ Within 6 months (2)
- ☐ Within a year (3)
- ☐ Within 3 years (4)
- ☐ More than 3 years (5)

In the past month, how frequently did you change your face mask?

- ☐ More than once a day (5)
- ☐ Daily (4)
- ☐ Every other day (3)
- ☐ Every 3 day (2)
- ☐ More than 3 days per piece (1)

Developed questionnaire					
**5 = Strongly agree 4 = Agree 3 = Mediocre 2 = Disagree 1 = Strongly disagree**	**5**	**4**	**3**	**2**	**1**
**I agree with the following statements**					
1. Currently, I am willing to wear a face mask					
2. I am willing to spend money on face masks					
3. I always suggest that others wear masks					
4. I am willing to wear a mask, despite the official announcement not to enforce mask wearing					
5. I always bring a mask every time I go outside					
6. I am familiar with mask-wearing					
7. I believe that COVID-19 is still spreading throughout my community					
8. I believe that COVID-19 can reemerge in the future					
9. I still feel anxious about being infected with COVID-19					
10. I feel anxious or disgusted when someone not wearing a mask gets close to me					
11. I wear a mask to protect myself from air pollution					
12. I wear a mask to protect myself against other respiratory infections apart from COVID-19					
13. I trusted that Andrographis paniculata could effectively prevent COVID-19, so I reduced my mask-wearing.					
14. I trusted that practices such as eating cooked food, using serving spoons, and washing hands could effectively prevent COVID-19, so I reduced my mask-wearing.					
15. I trusted that cloth face masks could effectively prevent COVID-19, so I reduced my mask-wearing					
16. I believe in the effectiveness of face masks for COVID-19 protection.					
17. I believe that mask-wearing helps reduce the spread of COVID-19					
18. Wearing a face mask improves my self-esteem					
19. I believe that using a face mask makes me look better					
20. I feel uncomfortable when wearing a mask					
21. Wearing a mask impairs my work productivity					
22. Wearing a mask can be an obstacle to communication					
23. I wear a face mask because it is a social measure.					

24. I wear a face mask because others are also wearing them.					
25. I wear a face mask to conceal my poor oral hygiene					
26. I don’t have enough high-quality face masks to meet the standard.					
27. The high price is affecting my decision to purchase a face mask.					
28. I don’t have enough money to afford using a face mask daily.					
29. I think that buying face masks increases my financial burden.					
